# Evaluation of Binders in Twin-Screw Wet Granulation

**DOI:** 10.3390/pharmaceutics13020241

**Published:** 2021-02-09

**Authors:** Claudia Köster, Sebastian Pohl, Peter Kleinebudde

**Affiliations:** Institute of Pharmaceutics and Biopharmaceutics, Heinrich Heine University, Universitätsstr. 1, 40225 Duesseldorf, Germany; claudia.koester@hhu.de (C.K.); sebastian.pohl@hhu.de (S.P.)

**Keywords:** twin-screw wet granulation, granule size distribution, particle size, tabletability

## Abstract

The binders povidone (Kollidon 30), copovidone (Kollidon VA64), hypromellose (Pharmacoat 606), and three types of hyprolose (HPC SSL-SFP, HPC SSL, and HPC SL-FP) were evaluated regarding their suitability in twin-screw wet granulation. Six mixtures of lactose and binder as well as lactose without binder were twin-screw granulated with demineralized water at different barrel fill levels and subsequently tableted. A screening run with HPC SSL determined the amount of water as an influential parameter for oversized agglomerates. Subsequent examination of different binders, especially Kollidon 30 and Kollidon VA64 resulted in large granules. All binders, except Pharmacoat 606, led to a reduction of fines compared to granulation without a binder. The molecular weight of applied hyproloses did not appear as influential. Tableting required an upstream sieving step to remove overlarge granules. Tableting was possible for all formulations at sufficient compression pressure. Most binders resulted in comparable tensile strengths, while Pharmacoat 606 led to lower and lactose without a binder to the lowest tensile strength. Tablets without a binder disintegrated easily, whereas binder containing tablets of sufficient tensile strength often nearly failed or failed the disintegration test. Especially tablets containing Pharmacoat 606 and HPC SL-FP disintegrated too slowly.

## 1. Introduction

While processes were performed batchwise in the past, the interest of the pharmaceutical industry in continuous manufacturing has increased in recent years. Batchwise manufacturing is characterized by separate performance of process steps while continuous manufacturing does not include disruptions of the process, as all manufacturing units are directly connected; while the initial material is added at the first unit, the final product is simultaneously discharged at the last one [1]. The latter affords the opportunity to use 24 h automatic product lines, a reduction of development time caused by lack of challenging upscaling procedures, as well as a decrease in labor costs, device costs, and processing time [2,3,4]. Moreover, continuous manufacturing features advantages with regard to quality, flexibility, and safety [5]. Twin-screw wet granulation is a granulation method, which enables continuous manufacturing and thereby is an auspicious manufacturing technique. Many studies dealt with the influence of different process parameters like liquid-to-solid ratio (L/S) or the barrel fill level affected by the variation of powder feed and screw speed [2,6,7,8,9,10,11,12]. Moreover, equipment parameters like the influence of different screw elements and their arrangement were frequently examined [13,14,15,16]. Another important factor is the composition of the used powder blend. Dependent on the properties of applied substances, the addition of a binder is necessary to facilitate granulation. The choice of binder influences granule properties [17]. Since the manufacturing of granules as an intermediate product of tablet preparation is common in the pharmaceutical industry, binders also influence the properties of obtained tablets. However, if the material is soluble in the granulation liquid (e.g., lactose), granulation without binder addition is possible [4].

Many binders are applicable to granulation processes. In the best case, they generate a cohesive network between the formulation ingredients [18]. One group of established binders are semisynthetic products of cellulose, for example, hyprolose (HPC) or hypromellose (HPMC). Likewise, polymers and copolymers of *N*-vinyl pyrrolidone are frequently used binders. Recently an extensive comparison of the impact of different binders on twin-screw granules was published [18]. However, there was no examination of resulting tablets as some groups did it in the context of roll compaction/dry granulation [19,20]. Therefore, the aim of this study was an investigation of the impact of six binders and the absence of binders on the properties of granules and tablets. In order to compare and evaluate their suitability for twin-screw wet granulation, granules and tablets were examined.

## 2. Materials and Methods

### 2.1. Materials

Table 1 gives an overview of used binders. Different types of hyprolose were used. Table 2 lists their molecular weight and particle size. Additional applied substances were lactose monohydrate (Granulac^®^ 200, Meggle, Wasserburg am Inn, Germany) as filler, silicium dioxide (Aerosil^®^ 200 Pharma, Evonik, Hanau-Wolfgang, Germany) to improve flow properties, and magnesium stearate (LIGAMED^®^ MF-2-V, Peter Greven Nederland C.V., Venlo, The Netherlands) as a lubricant for tableting.

### 2.2. Methods

#### 2.2.1. Preparation of Granules

Lactose and binder were applied in a ratio of 95:5 (*w*/*w*) and blended with 0.2% (*w*/*w*) silicium dioxide for 20 min at 25 rpm (LM 40, L.B. Bohle Maschinen und Verfahren, Ennigerloh, Germany). The batch size totaled 6–12 kg, depending on the number of runs. Before blending, silicium dioxide was sieved through a 355 µm sieve. The water content of obtained powder blend was determined threefold (Sartorius Moisture Analyzer MA100, Sartorius, Göttingen, Germany). Depending on that, the flow rate of the granulation liquid water was chosen to achieve the desired L/S.

Granulation was performed on a 16 mm twin-screw granulator (Pharma 16, Thermo Fisher Scientific, Waltham, MA, USA), which exhibited a barrel with a length-to-diameter ratio (L/D) of 40:1. The screw configuration included long pitch conveying elements (LPCE), short pitch conveying elements (SPCE), and kneading elements (KE) at a 60° stagger angle. Their arrangement in flow direction was 4D LPCE > 4D SPCE > 1.25D (5) KE > 10D SPCE > 1.5D (6) KE > 19.5D SPCE. Screws had an L/D of 41:1 to avoid material densification and adhesion at the outlet of the barrel. The powder blend was fed into the barrel by a twin-screw loss-in-weight feeder (K-ML-KT20, K-Tron, Stuttgart, Germany). Demineralized water as granulation liquid was added using a micro annular gear pump (MZR 7205, HNP-Mikrosysteme, Schwerin, Germany) and an injector nozzle with an inner diameter of 0.75 mm. The injector nozzle was placed at the SPCE before the first kneading block above one of the screws. Granulation was performed at a barrel temperature of 30 °C (STL 1-0-B5/10-TK6, Single Temperiertechnik, Hochdorf, Germany).

Samples of about 500 g were taken after the process equilibrium had been reached. The granules were dried in a compartment drier (kelvitron^®^ t, Heraeus Instruments, Hanau, Germany) at 30 °C until residual moisture lower than 1% was achieved. Afterward, they were stored in closed plastic bags.

#### 2.2.2. Determination of Useful Process Settings

During a screening experiment with HPC SSL as a binder, different process conditions were examined in order to identify suitable settings for the L/S and the specific feed load (SFL), a surrogate for the barrel filling degree. Whereas the L/S was described as the ratio of liquid feed (g/min) and powder feed (g/min), SFL was defined as the ratio of powder feed (g/min) and screw speed (1/min) [7]. A 3^2^ experimental design was applied to determine suitable process settings whereby the center point was performed three times. Table 3 depicts implemented process settings.

#### 2.2.3. Granulation with Different Binders

Granulation using various binders and without binder was performed at SFL of 0.350 g, 0.157 g, and 0.080 g at L/S of 0.075, which corresponds to No. 1, 2, and 3 in Table 3.

#### 2.2.4. Tableting

Granules manufactured at SFL 0.080 g and L/S 0.075 were used for tableting because these settings produced most granules, which accomplished x90% smaller than 1500 µm, which was set as threshold level (Section 2.2.6). A sieve of 1000 µm was used to remove oversized agglomerates. Obtained granules were blended at 49 rpm with silicium dioxide for 8 min and with magnesium stearate for a further 2 min (98:1:1 *w*/*w*, Turbula^®^ mixer T2F, Willy A. Bachofen, Muttenz, Switzerland). Round tablets with a diameter of 8 mm were manufactured on a Pressima (IMA Kilian, Köln, Germany) at a turret speed of 10 rpm using two flat-faced punches (Ritter Pharma-Technik, Stapelfeld, Germany). Four pressures were applied (50 MPa, 100 MPa, 200 MPa, 300 MPa). The height mode was used for tableting, where the lower punch set the die height, hence the filling quantity and tablet weight. Test tablets were manufactured to verify the intended tablet weight of 200 ± 2 mg and to determine the target position of the lower punch. Afterward, the end position of the upper punch was modified to reach the intended compression force.

#### 2.2.5. Dynamic Vapor Sorption of Base Material

The dynamic vapor sorption was examined with a vapor sorption analyzer (SPS 11, ProUmid, Ulm, Germany). Pure binders, as well as powder blends of lactose, binder and silicium dioxide, were examined. Initially, samples were dried at 0% relative humidity. In the following, relative humidity was increased in steps of 10% up to 80%. The relative humidity was automatically increased when mass changed less than 0.01% in 30 min, but not before 60 min after the last increase of relative humidity and latest after 48 h, independent of mass changes. The measurement was performed once for every sample at 30 °C.

#### 2.2.6. Characterization of Granules

At first, the samples were prepared with a sample divider (Sample divider PT and PT 100, both Retsch, Haan, Germany) and three sub-samples were investigated. The sub-samples included at least 1,000,000 particles. Using Feret diameter, the particle size distribution (PSD), x10%, x25%, x50%, x75% and x90% quantiles of the volume distribution and span of each sub-sample were examined using dynamic image analysis (CAMSIZER XT, Retsch Technology, Haan, Germany). Span was defined as (Equation (1)):

x90% [µm] − x10% [µm])/x50% [µm](1)

A target value of x90% < 1500 µm was defined. Moreover, fines were defined as particles < 200 µm, and particles > 2000 µm were classified as oversized. For measurement preparation, samples were sieved through a 2800 µm sieve. Sieved large particles were removed to avoid blockage of the instrument. 

#### 2.2.7. Characterization of Tablets

The uniformity of mass was investigated following European Pharmacopoeia (Ph.Eur.) 2.9.5 [22]. The coefficient of variation (CV, ratio of standard variation and mean) was calculated additionally.

Crushing force, diameter, and thickness were measured (Smart Test 50, Pharmatron Dr. Schleuniger, Thun, Switzerland) after storage of at least 20 h. Tensile strength was calculated applying hardness (H), thickness (T), and diameter (D) according to Equation (2) [23] and correlated to compaction forces of particular tablets. A tensile strength of 2 MPa was defined as sufficient to withstand stress during normal handling [24].

(2) $$Tensile\;strength\;\left[\mathrm{MPa}\right]\;=\;\frac{2\;\times \;H\;\left[N\right]}{\pi \;\times \;T\;\left[\mathrm{mm}\right]\;\times \;D\;\left[\mathrm{mm}\right]}$$

Disintegration test of tablets was performed using Pharma Test (PTZ Auto 1EZ, Pharma Test Apparatebau, Hainburg, Germany) according to Ph.Eur. 2.9.1 [22]. Disintegration time was stopped manually.

## 3. Results and Discussion

### 3.1. Dynamic Vapor Sorption

**Figure 1:**
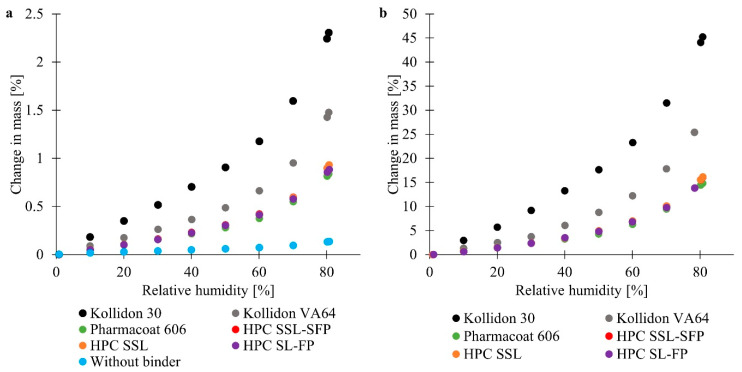
Dynamic vapor sorption of (**a**) powder blends containing different binders or no binder (*n* = 1) and (**b**) of different pure binders (*n* = 1). HPC SSL-SFP and HPC SSL are overlapping. HPC: hyprolose.

The blend of lactose and silicium dioxide barely changed in mass independently of the relative humidity (Figure 1a). Therefore, the binders were crucial for water sorption. The results of powder blends and pure binders exhibited the same trends regarding the order of applied binders (Figure 1a,b). The water uptake of pure binders equated to about twentyfold the water uptake of blends and thus corresponded to the amount of binder. Cellulose-based polymers led to lower changes in mass, which were comparable between the HPC types and HPMC. However, HPCs might absorb little more water than HPMC at high relative humidity, e.g., HPC SSL-SFP presented a change in mass of 15.5% at a relative humidity of 80.2%, where the weight of Pharmacoat 606 increased 14.5%. Even after a closer look, HPC SSL-SFP and HPC SSL did not present differences, but HPC SL-FP showed a slightly lower water uptake than HPCs of lower molecular weight (change in mass of 10.1%, 10.1%, and 9.8%, respectively, at 70.1% relative humidity). Nevertheless, mentioned differences between the cellulose-based polymers were only minor, and measurement was only performed once for pure binders and once for blends. The longer cellulose chains of HPC SL-FP might be less flexible and permit less water uptake than the shorter cellulose chains of HPC SSL. The lower hygroscopicity of HPMC might correlate with the methyl substituent. Kollidon 30 was most hygroscopic and presented a change in mass up to more than 45% (related to the mass of pure Kollidon 30) at about 80% relative humidity (Figure 1b). The second highest water uptake was observed for Kollidon VA64.

Examination of dynamic vapor sorption of PVP K29/30, HPC EXF, and HPMC E5 (comparable molecular weights to Kollidon 30, HPC SL-FP, and Pharmacoat 606) led to similar trends [25]: PVP K29/30 exhibited the highest and fastest water uptake, water sorption of HPC EXF and HPMC E5 was comparable. The residual ordered structure of the cellulose-based polymers, as well as their more rigid chains, resulted in lower water uptake. Moreover, a longer residence at the gel state of the cellulosic polymers than of PVP was observed [25].

### 3.2. Determination of Useful Process Settings

**Figure 2:**
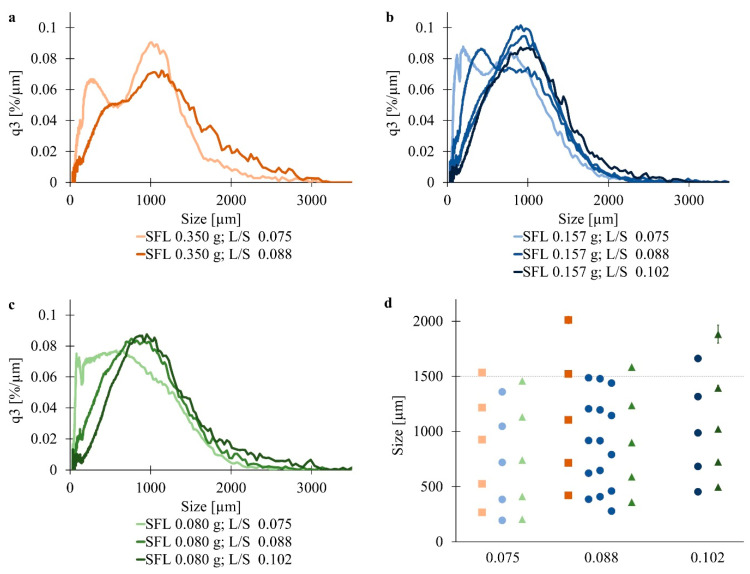
Density distribution of (**a**) SFL 0.350 g, (**b**) SFL 0.157 g and (**c**) SFL 0.080 g at different L/S (*n* = 3; $\bar{x}$) and (**d**) x values (x10%, x25%, x50%, x75%, x90%) of SFL 0.350 g (■, brown), SFL 0.157 g (●, blue) and SFL 0.080 g (▲, green) at different L/S (darker nuance with increasing L/S; *n* = 3; $\bar{x}$ ± sd).

At all settings, particle growth was obtained, but to a different extent (Figure 2). Granulation at SFL 0.350 g and L/S 0.102 led to an extensive increase, and oversized agglomerates were received that were not measurable with Camsizer. It was necessary to remove the largest oversized particles at all three sub-samples, 56%, 61%, and 63% (*w*/*w*), by sieving through 2800 µm sieve to avoid blockage of the measurement instrument. Consequently, results would only describe less than half of the obtained particles and were, therefore, not considered in the following.

**Figure 3:**
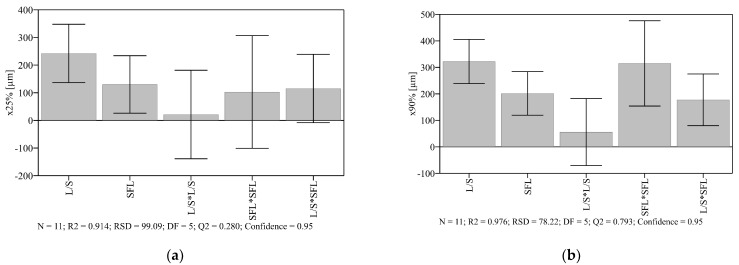
Coefficient plot of (**a**) x25% and (**b**) x90% of HPC SSL granules.

SFL, as well as L/S, presented an influence on the particle size distribution of granules. At SFL 0.350 g, L/S 0.075 led to a bimodal PSD, whereas L/S 0.088 led to a rather monomodal distribution (Figure 2a). Higher liquid contents reduced the fraction of particles smaller than 500 µm. Besides the reduction of fines and small particles, L/S 0.088 resulted in more particles larger than 1300 µm compared to L/S 0.075. However, obtained granules exceeded the x90% limit value, independently of the L/S, when granulation was performed at SFL 0.350 g (Figure 2d). The formation of partly high fractions of large agglomerates was also described in several studies, where granulation was performed with different powder blends, screw configurations, and barrel fill level [6,26,27,28,29,30]. At SFL 0.157 g and SFL 0.080 g, L/S 0.075 resulted in trimodal and bimodal PSD, respectively, whereas L/S 0.088 and L/S 0.102 resulted in a monomodal PSD (Figure 2b,c). Especially at SFL 0.080 g, L/S 0.102 presented a right-shifted curve compared to L/S 0.088. Contrary to SFL 0.350 g, x90% limit value was not exceeded at L/S 0.075 when granulation was performed at lower SFLs (Figure 2d). Repetition of center point settings led to divergent PSDs, although process data were comparable. Further increase of the liquid amount to L/S 0.102 led to x90% larger than 1600 µm (SFL 0.157 g) and 1800 µm (0.080), respectively. Observations were clarified by statistical analysis with Modde (Figure 3a,b). A higher L/S significantly increased all investigated x values. Caused by a higher amount of water, more powder particles could be wetted or solved, more liquid bridges were formed, and therefore agglomeration was enhanced. An increase of granule sizes at higher L/S was also observed in former studies of twin-screw granulation [9,11,29,31,32]. Moreover, bimodal PSDs were observed at lower L/S. Assumptions comprise unequal wetting or lower liquid availability on the particle surface, which led to less coalescence of larger particles [6,7,9].

Comparing different SFLs at the same L/S, SFL 0.350 g led to fewer fines and the most oversized granules (Figure 2a–d). Analysis with Modde revealed a significant increase of all examined x values at rising SFL (Figure 3a,b). A higher barrel fill might result in more interactions between the particles and therefore increased formation of agglomerates [8]. The assumption that a higher barrel fill leads to increased shear forces and abrasion, which, in turn, results in more fines [33], could not be confirmed with this study. Congruent to their results, at lower fill level, less abrasion intensified back mixing, and less packaging of primary particles, and therefore larger agglomerates were described [26]. As opposed to this, another group found fewer oversized particles at higher screw speed and, therefore, lower barrel fill [29]. Further studies detected an influence of SFL in a positive or negative way or even no influence [28,34,35]. Nevertheless, the material residence time might also have an effect on the PSD. Especially the screw speed was found to be decisive on that [36]. Although no residence time measurements were performed, it can be assumed that lower screw speeds caused longer residence times of the material inside the barrel. Augmented solution and particle growth would be encouraged, and therefore larger agglomerates would be obtained. However, in this study, residence time was not examined, and therefore, an influence could not be assumed without further study. Nevertheless, the inconsistent results of the above-mentioned studies suggest that barrel fill does not influence the PSD solely, which is also indicated by the interaction of L/S and SFL (Figure 3b).

Considering the x90% limit value, granulation at L/S 0.075 seemed to be the most promising setting. Because of the less predictable influence of SFL, established SFLs were performed in the following study.

### 3.3. Granulation with Different Binders at L/S 0.075

**Figure 4:**
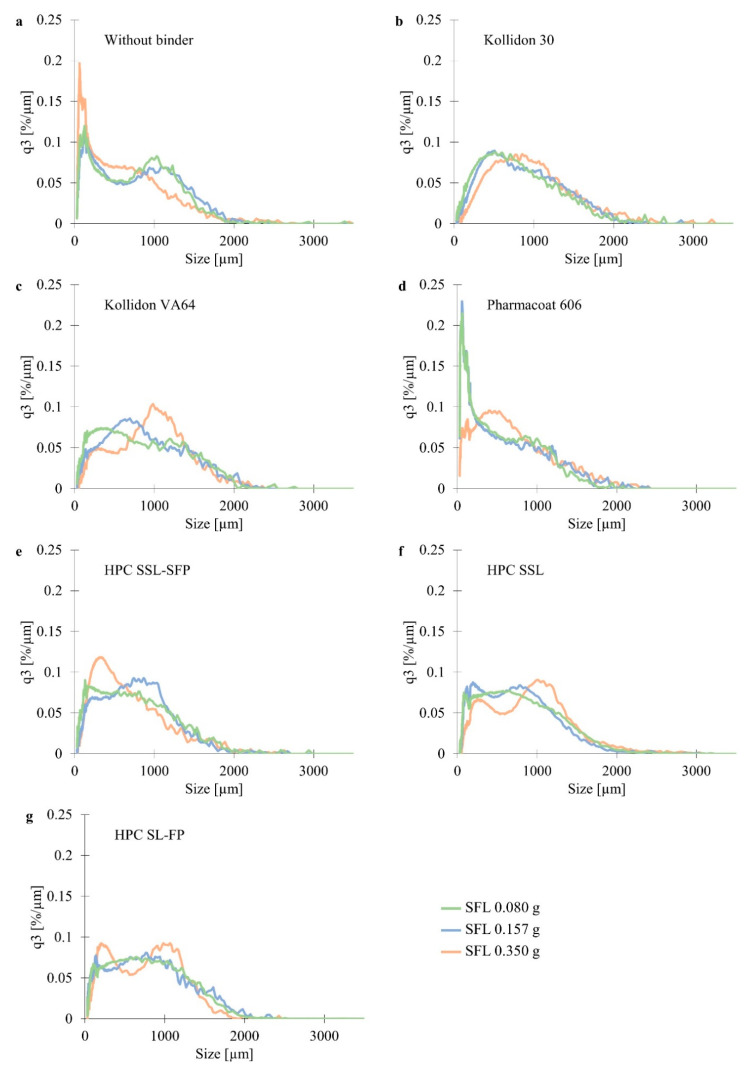
Density distribution of granules manufactured (**a**) without binder, (**b**) with Kollidon 30, (**c**) Kollidon VA64, (**d**) Pharmacoat 606, (**e**) HPC SSL-SFP, (**f**) HPC SSL, (**g**) HPC SL-FP at L/S 0.075 (*n* = 3; $\bar{x}$).

**Figure 5:**
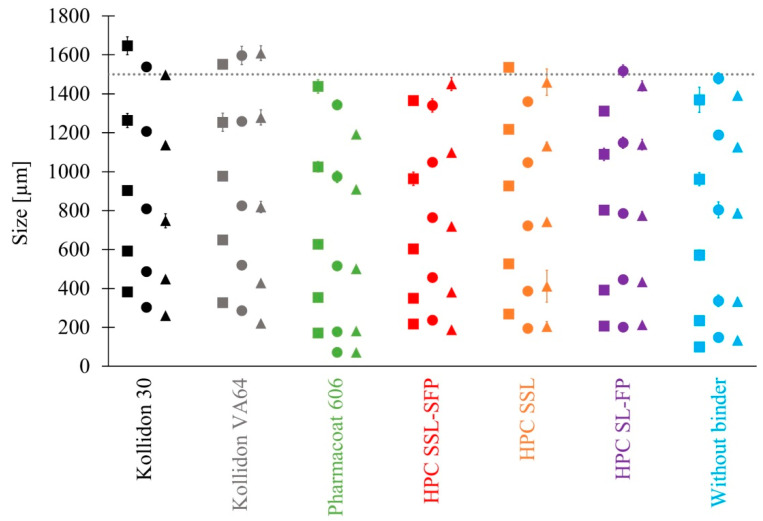
X values (x10%, x25%, x50%, x75%, x90%) of granules manufactured with different binders at SFL 0.350 g (■), SFL 0.157 g (●) and SFL 0.080 g (▲) at L/S 0.075 (*n* = 3; $\bar{x}$ ± sd).

Comparing granules with and without binder, a reduction of fines was observed when a binder was added (Figure 4a–g). However, Pharmacoat 606 could almost not decrease the fraction of fines in relation to binderless granules. x50%, x75%, and x90% of binderless granules were comparable to some binder containing granules, for example, HPC SSL-SFP (Figure 5). Granulation without a binder did not lead to a distinct reduction of large particles. This would be a consequence of less binding capacity in the absence of a binder. The size of large particles was small enough to meet the requirements at two of three SFLs, but the usage of some binders also resulted in acceptable x90%.

The influence of SFL depended on the binder (Figure 4a–g). For all granules, SFL 0.350 g differed from lower SFLs. Nevertheless, in the case of binderless granulation, SFL 0.350 g resulted in a higher fraction of fines. Granules without binder probably were less stable, and abrasion occurred at higher fills. This would also explain fewer large particles than at granulation with some binders, e.g., Kollidon 30. When granulation was performed with Kollidon 30, SFL had only a slight influence. An increase of SFL led to a right-shift of the PSD, but the shape was not influenced. Independently of the SFL, the PSD was monomodal. In contrast, SFL presented a more distinct impact in the case of Kollidon VA64. The global maximum of the PSD exhibited a right shift and an increase at higher SFL. So, the higher SFL might lead to an increase in particle interactions and increased formation of agglomerates. However, SFL barely impacted particles larger than 1300 µm. Granulating with Pharmacoat 606, the fraction of fines was distinctly influenced by the SFL. SFL 0.350 g reduced fines and increased particles at size range from 350 µm to 770 µm. Only the highest examined SFL seemed to be able to ensure enough interactions and, therefore, a reduction of fines. This observation differed from other binders, where lower SFL did not result in a comparable fraction of fines. HPC SSL-SFP as binder led to monomodal or nearly monomodal PSDs. Surprisingly, SFL 0.350 g resulted in most particles in the range of 200 µm to 600 µm. However, other powder compositions did not show this trend. The higher fill level might increase the abrasion of larger agglomerates resulting in smaller granules. SFL 0.080 g and SFL 0.157 g resulted in more particles between 600 µm and 1100 µm. The coarser powder quality of the chemical equivalent polymer, HPC SSL, presented bimodal (SFL 0.080 g and SFL 0.350 g) or trimodal (SFL 0.157 g) PSDs. Usage of a higher molecular weight (HPC SL-FP) led to bimodal PSDs. Bimodality was most distinct at SFL 0.350 g. The highest SFL presented more small particles than SFL 0.157 g and SFL 0.080 g, more particles at the size range of 1000 µm but fewer particles larger than 1500 µm. The reduction of fines at higher L/S, which was observed at different compositions, was also determined in the screening study (3.2) and might be a consequence of increased wetting of particles and development of liquid bridges, and in consequence, formation of agglomerates.

Moreover, residence time might be different at the SFLs because of various screw speed. Increased residence time at lower screw speed (higher SFL) could enhance the dissolution of powder particles and therefore support agglomeration. However, viscosities of binder solutions might also impact the residence time and, therefore, would explain the divergent influence of SFL dependent on the applied composition.

An increase of x values at higher SFLs, which was found to be significant in Section 3.2 (Figure 3a,b), was presented by Kollidon 30 (Figure 5). The effect was only determined at x10% and x25% when granulation was performed with Kollidon VA64, and standard deviations were considered. Pharmacoat 606 especially led to larger x values at SFL 0.350 g. By contrast, the highest SFL resulted in the lowest x values when granulation was performed without a binder. So, the influence of the SFL is apparently dependent on the composition.

Comparing the granules prepared with different binders, Kollidon 30 and Kollidon VA64 resulted in the largest granules (Figure 5). They always failed the x90% target. By contrast, Pharmacoat 606 and HPC SSL-SFP resulted in x90% < 1500 µm, independently of applied SFL. Other binders partly met the requirements. Many small particles, recognizable by a small x10%, were obtained with Pharmacoat 606 and without a binder. HPC SSL-SFP and HPC SSL, which only differ in the size of powder particles, resulted in comparable x values at SFL 0.157 g and SFL 0.080 g. By contrast, x values at SFL 0.350 g differed. HPC SSL led to larger ones. At the highest SFL, particle sizes of HPC SSL-SFP granules were even more similar to that of HPC SL-FP, which exhibits a higher molecular weight. Fine powder particles of HPC SSL-SFP might be able to reduce the fines more than coarser powder qualities. Caused by a larger surface, they might exhibit a faster solution. However, this expected reduction of fines was not observed. X10% values were not larger than the ones of HPC SSL. Comparing the HPCs, no clear influence of the molecular weight was identifiable. Dependent on the SFL, the higher molecular weight resulted in comparable values or larger x values. Latter was only observed at SFL 0.157 g. Similar to the current study, Stoyanov et al. (2018) compared HPCs with different molecular weights. However, they examined high-shear and fluid-bed granulation. In case of fluid-bed granulation, HPC SSL (molecular weight 40,000 [21]) resulted in smaller granules and more narrow PSD than the larger molecules of HPC SL (molecular weight 100,000 [21]) and HPC L (molecular weight 140,000 [21]), which presented the largest granules. The finer HPC qualities HPC SSL-SFP, HPC SL-FP, and HPC L-FP (same molecular weight as HPC SSL, HPC SL, and HPC L, respectively [21]) were used in high-shear granulation. Despite the different molecular weights of HPC SSL-SFP, HPC SL-FP, and HPC L-FP, comparable PSDs were received [37]. Reversed results were published concerning HPMCs [38,39]. Although both used foam granulation, same powder matrix, and HPMC types, Weatherley et al. (2013 [38]) received significantly more large particles with increasing molecular weight (*p* < 0.05) whereas Thompson et al. (2012 [39]) observed smaller and weaker granules with higher molecular weight. The influence on particle size was found to be not significant, but the influence on strength was. Therefore, the influence of the molecular weight of binders on PSDs of wet granules seemed not to be a general effect.

Other studies, which compared the influence of different binders on granules, partly showed the same trends. Comparison of Kollidon VA64 fine (finer quality of Kollidon VA64), Methocel E5 (comparable to Pharmacoat 606), and HPC SSL-SFP in high-shear granulation presented lowest x50% for granulation with HPMC [40] and thus the same result as the present study. They observed the largest x50% dependent on the amount of their granulation liquid water. Using an L/S of 1%, HPC SSL-SFP resulted in the highest x50%, using L/S 1.5%, Kollidon VA64 fine led to the largest x50%. The L/S was not varied in the present test series, and hence the influence of the amount of water could not be observed. Independently of the SFL, Kollidon VA64 always resulted in larger x50% than HPC SSL-SFP. The differences between the results of the studies might be caused by the different wet granulation technique and the binders, which were similar but not identical. Smaller particles with HPMC (Methocel E5 Premium LV) as binder compared to Kollidon 30 were also observed by further researchers, who performed high-shear granulation of lactose. However, an exchange of the filler to mannitol led to reversed results [41]. Thus, the effect of the binder also depends on the composition of the powder blend. Moreover, various PSDs of the compositions observed in the present study might be caused by differences in the wetting of the applied polymers, e.g., in water sorption.

Polymers must be dissolved to work as a binder. Therefore, it is helpful to know their water sorption (3.1). Kollidon 30 seemed to present a more pronounced water uptake than Kollidon VA64, which in turn, absorbed more water than HPCs and Pharmacoat 606. Furthermore, PVP stayed briefer at gel state than HPC and HPMC [25]. A more pronounced water uptake might explain the larger particles and greater reduction of fines with Kollidon 30 and Kollidon VA64. However, results of water sorption did not explain the high fraction of fine particles with Pharmacoat 606 compared to HPCs.

**Figure 6:**
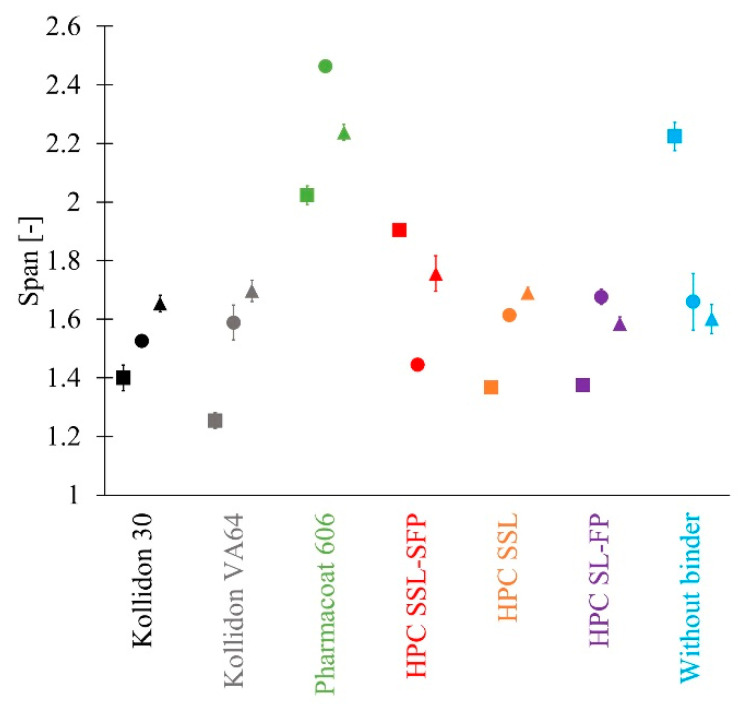
Span of size for granules manufactured with different binders at SFL 0.350 g (■), SFL 0.157 g (●) and SFL 0.080 g (▲) at L/S 0.075 (*n* = 3; $\bar{x}$ ± sd).

Span was sparsely influenced by the choice of binder (Figure 6). The addition of most binders resulted in a reduction of the span except Pharmacoat 606, which stood out in particular by a higher span of at least 2. At SFL 0.157 g and SFL 0.080 g, it was even higher than the span of granules without a binder. However, except SFL 0.350 g, the span of binderless granules was at a comparable range as the most binder containing granules. The SFL might also impact span. Kollidon 30, Kollidon VA64, and HPC SSL presented an increase of span at lower SFL. It is possible that the influence is inverted when no binder is used.

### 3.4. Characterization of Tablets

Mass variation of tablets was comparable in all cases, regardless of the binder used for granulation (Table 4). All batches passed the uniformity of mass (Ph.Eur. 2.9.5). In the case of HPC SSL-SFP and HPC SL-FP, one tablet of one applied pressure differed more than 7.5% from mean but none more than 15%. Overall, the coefficient of variation ranged between 0.85% and 4.46%. However, a sieving step was necessary to remove oversized agglomerates to achieve the above-described consistent weight.

**Figure 7:**
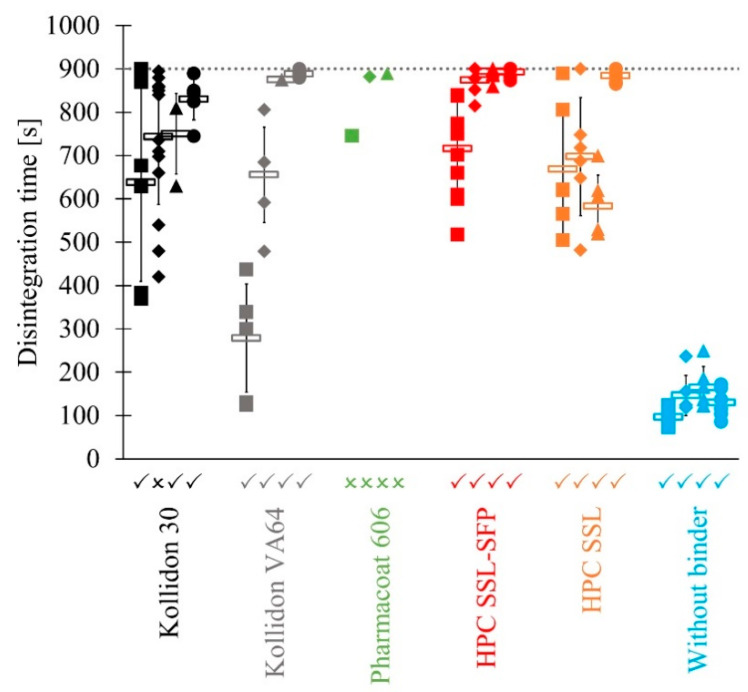
Disintegration time of passed (✓) and failed batches (🗴) of tablets manufactured at 50 MPa (■), 100 MPa (♦), 200 MPa (▲) and 300 MPa (●) including only passed tablets; single values and means (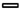; $\bar{x}$ ± sd).

Most of the examined tablets presented erosion rather than disintegration. This might be caused by the formation of a gel layer by the binder at the tablet surface. Therefore, penetration of water into the tablet was inhibited. Erosion resulted from the removal of the gel layer. Some tablets passed the disintegration test independently of applied pressure (Figure 7). Tablets manufactured with Kollidon VA64, HPC SSL-SFP, and HPC SSL as well as tablets without binder disintegrated within 15 min. Kollidon 30 led to tablets that passed the disintegration except the lot manufactured at 100 MPa. However, although disintegration tests were passed, disintegration needed nearly 900 s when compression was performed at 300 MPa, and a binder was used. By contrast, tablets without binder always disintegrated within 300 s at the maximum. The disintegration of tablets containing Pharmacoat 606 or HPC SL-FP mostly required more than 900 s and therefore failed. One tablet of each Pharmacoat 606 lot passed. In the case of HPC SL-FP, no tablet passed, which is why HPC SL-FP is not presented in Figure 7. The viscosity of generated gel layer might have been too high to enable the penetration of water into the tablet. HPC SL-FP possesses a higher molecular weight than HPC SSL. The lower molecular weight of HPC SSL resulted in a lower viscosity of the gel layer and, thus to faster disintegrating tablets. Examinations of water sorption (3.1) presented faster and more pronounced water sorption of Kollidon 30 and Kollidon VA64 than of HPCs and Pharmacoat 606. Moreover, HPC and HPMC stay at the gel state for a longer time than PVP before they changed into a solution [25]. Both observations could explain the slower disintegration of tablets containing Pharmacoat 606 and HPC SL-FP compared to Kollidon 30 and Kollidon VA64. However, the disintegration of tablets containing HPC SSL-SFP and HPC SSL was not always slower than that of tablets containing Kollidon 30 and Kollidon VA64, which could be assumed based on the results of water sorption.

Higher pressure led to increased densification of material and might slow disintegration. Means of Kollidon 30, Kollidon VA64, and HPC SSL-SFP presented this trend. This trend was especially pronounced for Kollidon VA64 as the binder. The copolymer led to the fastest disintegration of a binder containing tablets at an applied pressure of 50 MPa. Binderless tablets exhibited by far the fastest disintegration. Without a binder, no gel was formed that could have inhibited water permeation. Therefore, a mixture of disintegration and erosion was observed. Variation within one tablet lot indicated various manufacturing conditions, for example, differing applied pressure. For example, during compression of Kollidon 30 tablets, an applied pressure of 103.0 ± 12.1 MPa (CV = 11.8%) was measured. The CV was even 15.3% for the compression of Kollidon VA64 at 50 MPa. Various compression forces resulted in various densification and consequently could influence the disintegration time. Concerning all compositions, CV accounted for 9.8%. As mentioned in Section 2.2.4, tableting was performed with height mode. Unequal die fill would explain diverse compression forces. However, mass fluctuation, which would also result from unequal die fill, was not pronounced.

Comparing the influence of different binders, former studies observed a faster disintegration of HPMC containing tablets (Methocel E5 Premium LV) compared to tablets containing Kollidon 30 [40]. However, they performed high-shear granulation instead of twin-screw granulation and therefore achieved different granules. Additionally, the HPMC type was not identical to the present study. Methocel E5 Premium LV possesses a lower viscosity than Pharmacoat 606, and therefore disintegration would be less hampered by the formation of a gel layer. An examination of Kollidon 30, Kollidon VA64, HPC SSL-SFP, and HPC SL-FP in roll compaction (dry granulation) and tableting determined the quickest mean disintegration with Kollidon VA64 followed by HPC SL-FP, Kollidon 30, and HPC SSL-SFP [19]. Thus, there were analogies to the current study: Kollidon VA64 revealed the fastest mean disintegration time at 50 MPa, and Kollidon VA64, as well as Kollidon 30 tablets, disintegrated faster than tablets that contained HPC SSL-SFP. The structure of their tablets might be different because they used a dry granulation method and therefore received various granules. However, compression of granules produced with various granulation techniques led to different tablets caused by different PSD and surface structure of the granules [42].

**Figure 8:**
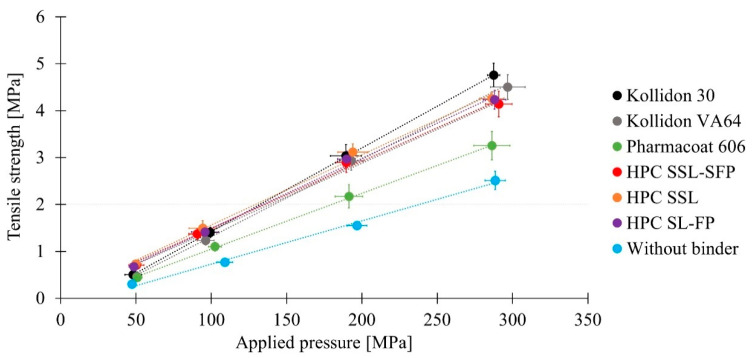
Tensile strength of tablets manufactured with different binders (*n* = 10; $\bar{x}$ ± sd).

Using a higher applied pressure, stronger tablets were received caused by greater densification (Figure 8). The absence of a binder led to the lowest tensile strength independent of applied pressure. Pharmacoat 606 also resulted in tablets with lower tensile strength than other binders. Kollidon 30, Kollidon VA64, HPC SSL-SFP, HPC SSL, and HPC SL-FP presented comparable tensile strengths. Concerning only mean tensile strength, Kollidon 30 led to the highest tensile strength at highest applied pressure and showed as well as Kollidon VA64 a great increase of tensile strength with rising applied pressure. It has been described in the literature that tablets containing different HPC types presented comparable tensile strengths when applied pressures were below 113 MPa [37]. At higher pressures, HPCs with lower molecular weight led to more resistant tablets. Authors supposed the correlation of strength and molecular weight to higher deformability of shorter HPC SSL polymer [37]. Applying dry granulation, higher tablet tensile strengths at decreasing molecular weight of HPCs were also described in a comparison of HPC SSL-SFP, HPC SSL, and HPC L-FP (molecular weights of 40,000, 40,000, and 140,000, respectively). The differences in tablet tensile strengths increased at rising compaction pressures [20]. However, Mangal et al. [20] used roll compaction for granulation, and therefore compressed granules differed from the present study. Divergence in granulation technique would also explain the different results to another dry granulation study, where HPC SSL-SFP revealed the highest tensile strength followed by Kollidon VA64 and HPC SL-FP, which had overlapping standard deviations and lowest tensile strength was observed for Kollidon 30 [19]. The low tensile strength of HPMC (Methocel E5 Premium LV) tablets was also described in comparison with Kollidon 30; however, the variation was not significant [41].

**Figure 9:**
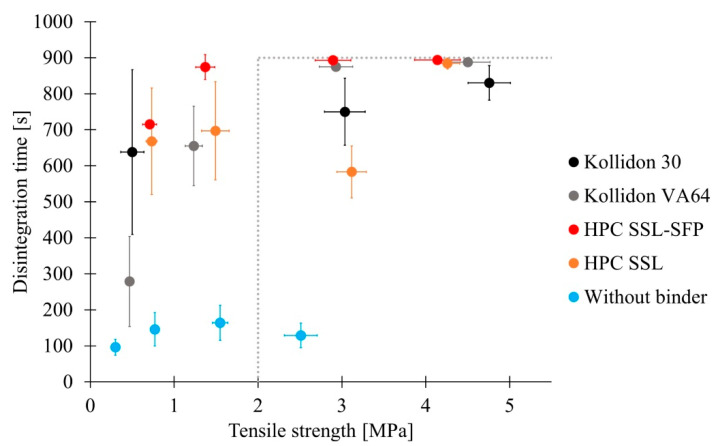
Quality of tablets including disintegration time and tensile strength including tablets which passed disintegration test (disintegration $\bar{x}$ ± sd; tensile strength *n* = 10, $\bar{x}$ ± sd).

Implying both examined quality criteria, where differences were determined, revealed that some binders, but also an absence of binder, could lead to acceptable tablets (Figure 9). Working without a binder required higher applied pressure to achieve sufficient tensile strengths but had the advantage to disintegrate fast without any problems, even without disintegrant. By contrast, tablets that contained binder and exhibited sufficient tensile strengths often did not disintegrate within 900 s or were close to the limit. A disintegrant might solve this problem. In this study, best results were achieved without binder and compression at 300 MPa, with Kollidon 30 at 200 MPa and 300 MPa, as well as with HPC SSL at 300 MPa.

## 4. Conclusions

Granule and, therefore, tablet properties are influenced by different conditions. Consequently, examination at different process settings is necessary to draw a conclusion about the general influences of different types of binders. The present study examined six different binders as well as the absence of binders in the context of twin-screw wet granulation and subsequent tableting. An increase of L/S led to particle growth. The influence of SFL was not that obvious. Only in some cases, a higher SFL resulted in larger granules, albeit the influence might be opposed in binderless granulation. Granulation of pure lactose was possible and resulted in acceptable granules and tablets when high pressures were applied during tableting. Binders often reduced fines. However, granulation with Pharmacoat 606 resulted in a fraction of fines comparable to or even higher than binderless granulation. Kollidon 30 and Kollidon VA64 led to larger granules than other binders. Examining different HPC types, a clear influence of molecular weight or powder particle size was not verifiable. Oversized granules, which were received with every binder but also without binder, were problematic for tableting and required a sieving step to ensure consistent tablet weights. Tablets of all compositions could be manufactured and exhibited sufficient tensile strengths above 200 MPa (binder containing tablets) or 240 MPa (binderless tablets) pressure application. Pharmacoat 606 tablets stood out by lower tensile strengths than other binder containing tablets but higher tensile strengths than binderless tablets. Disintegration was stated to be a problem when a binder was used. The addition of a disintegrant might ensure faster disintegration. Imagining continuous manufacturing processes, it is necessary to find granulation conditions, which inhibit the formation of oversized granules or integrate a continuous sieving step between granulation and tableting. However, the results of this study might not be transferable to less soluble powder compositions where the influence of the binder might be more important. Therefore, more investigations on different materials are required. Moreover, the usage of further twin-screw granulators seems to be vital and interesting for a better distinction of the suitability of different binders for twin-screw granulation.

## Figures and Tables

**Table 1:** Applied binders.

Product Name	Substance	Supplier	Name in Study
Kollidon^®^ VA64	Copovidone	BASF, Ludwigshafen, Germany	Kollidon VA64
Kollidon^®^ 30	Povidone	BASF, Ludwigshafen, Germany	Kollidon 30
NISSO HPC SSL SFP	Hyprolose	Nippon Soda, Tokyo, Japan	HPC SSL-SFP
NISSO HPC SL-FP	Hyprolose	Nippon Soda, Tokyo, Japan	HPC SL-FP
NISSO HPC SSL	Hyprolose	Nippon Soda, Tokyo, Japan	HPC SSL
Pharmacoat^®^ 606	Hypromellose	ShinEtsu, Tokyo, Japan	Pharmacoat 606

**Table 2:** Differences between applied hydroxypropyl cellulose types [21].

Name	Molecular Weight	Particle Size
HPC SSL-SFP	40,000	330 mesh Pass
HPC SSL	40,000	100 mesh Pass
HPC SL-FP	100,000	100 mesh Pass

**Table 3:** Experimental design.

No.	L/S (-)	SFL (g)	Screw Speed (1/min)	Powder Feed Rate (g/min)	No. Randomized Order
1	0.075	0.350	200	70	8
2		0.157	350	55	7
3		0.080	500	40	2
4	0.088	0.350	200	70	4
5		0.157	350	55	1
6					3
7					11
8		0.080	500	40	6
9	0.102	0.350	200	70	5
10		0.157	350	55	9
11		0.080	500	40	10

Achieved data were analyzed with Modde Pro 12.1 (Umetrics, Umeå, Sweden). SFL: specific feed load; L/S: liquid-to-solid ratio

**Table 4:** Mass of tablets compressed at various applied pressures (*n* = 20).

Used Binder	50 MPa		100 MPa		200 MPa		300 MPa	
	$\bar{x}$ ± sd (mg)	CV (%)	$\bar{x}$ ± sd (mg)	CV (%)	$\bar{x}$ ± sd (mg)	CV (%)	$\bar{x}$ ± sd (mg)	CV (%)
Kollidon 30	194.84 ± 3.21	1.65	200.91 ± 3.82	1.90	197.46 ± 2.63	1.33	199.77 ± 3.75	1.88
Kollidon VA64	200.39 ± 4.68	2.33	199.67 ± 2.85	1.43	202.01 ± 2.23	1.10	204.75 ± 2.63	1.28
Pharmacoat 606	208.89 ± 2.51	1.20	201.59 ± 3.30	1.64	198.89 ± 4.53	2.28	202.37 ± 4.64	2.29
HPC SSL-SFP	205.68 ± 3.12	1.51	200.23 ± 4.00	2.00	192.68 ± 7.42	3.85	192.23 ± 5.04	2.62
HPC SSL	207.90 ± 4.20	2.02	202.56 ± 2.61	1.29	201.46 ± 4.46	4.46	198.09 ± 3.42	1.72
HPC SL-FP	200.50 ± 5.00	2.49	195.22 ± 5.83	2.99	200.84 ± 4.92	2.45	196.71 ± 5.90	3.00
Without binder	201.99 ± 1.72	0.85	201.63 ± 2.46	1.22	202.13 ± 2.51	1.24	197.92 ± 4.54	2.30

## Data Availability

The data presented in this study are available on request from the corresponding author.

## References

[1] Roggo Y., Pauli V., Jelsch M., Pellegatti L., Elbaz F., Ensslin S., Kleinebudde P., Krumme M. (2020). Continuous manufacturing process monitoring of pharmaceutical solid dosage form: A case study. J. Pharm. Biomed. Anal..

[2] Keleb E. (2004). Twin screw granulation as a simple and efficient tool for continuous wet granulation. Int. J. Pharm..

[3] Fonteyne M., Vercruysse J., De Leersnyder F., Van Snick B., Vervaet C., Remon J.P., De Beer T. (2015). Process analytical technology for continuous manufacturing of solid-dosage forms. TrAC Trends Anal. Chem..

[4] Keleb E.I., Vermeire A., Vervaet C., Remon J. (2002). Continuous twin screw extrusion for the wet granulation of lactose. Int. J. Pharm..

[5] Pauli V., Kleinebudde P., Krumme M. (2020). Predictive model-based process start-up in pharmaceutical continuous granulation and drying. Pharmaceutics.

[6] El Hagrasy A., Hennenkamp J., Burke M., Cartwright J., Litster J.D. (2013). Twin screw wet granulation: Influence of formulation parameters on granule properties and growth behavior. Powder Technol..

[7] Lute S.V., Dhenge R.M., Salman A. (2018). Twin screw granulation: An investigation of the effect of barrel fill level. Pharmaceutics.

[8] Meier R., Moll K.-P., Krumme M., Kleinebudde P. (2017). Impact of fill-level in twin-screw granulation on critical quality attributes of granules and tablets. Eur. J. Pharm. Biopharm..

[9] Verstraeten M., Van Hauwermeiren D., Lee K., Turnbull N., Wilsdon D., Ende M.A., Doshi P., Vervaet C., Brouckaert D., Mortier S.T. (2017). In-depth experimental analysis of pharmaceutical twin-screw wet granulation in view of detailed process understanding. Int. J. Pharm..

[10] Gorringe L., Kee G., Saleh M., Fa N., Elkes R. (2017). Use of the channel fill level in defining a design space for twin screw wet granulation. Int. J. Pharm..

[11] Portier C., Pandelaere K., Delaet U., Vigh T., Di Pretoro G., De Beer T., Vervaet C., Vanhoorne V. (2020). Continuous twin screw granulation: A complex interplay between formulation properties, process settings and screw design. Int. J. Pharm..

[12] Portier C., Pandelaere K., Delaet U., Vigh T., Kumar A., Di Pretoro G., De Beer T., Vervaet C., Vanhoorne V. (2020). Continuous twin screw granulation: Influence of process and formulation variables on granule quality attributes of model formulations. Int. J. Pharm..

[13] Djuric D., Kleinebudde P. (2008). Impact of screw elements on continuous granulation with a twin-screw extruder. J. Pharm. Sci..

[14] Thompson M., Sun J. (2010). Wet granulation in a twin-screw extruder: Implications of screw design. J. Pharm. Sci..

[15] Vercruysse J., Díaz D.C., Peeters E., Fonteyne M., Delaet U., Van Assche I., De Beer T., Remon J.P., Vervaet C. (2012). Continuous twin screw granulation: Influence of process variables on granule and tablet quality. Eur. J. Pharm. Biopharm..

[16] Rahimi S.K., Paul S., Sun C.C., Zhang F. (2020). The role of the screw profile on granular structure and mixing efficiency of a high-dose hydrophobic drug formulation during twin screw wet granulation. Int. J. Pharm..

[17] Becker D., Rigassi T., Bauer-Brandl A. (1997). Effectiveness of binders in wet granulation: A comparison using model formulations of different tabletability. Drug Dev. Ind. Pharm..

[18] Vandevivere L., Denduyver P., Portier C., Häusler O., De Beer T., Vervaet C., Vanhoorne V. (2020). Influence of binder attributes on binder effectiveness in a continuous twin screw wet granulation process via wet and dry binder addition. Int. J. Pharm..

[19] Arndt O.-R., Kleinebudde P. (2018). Influence of binder properties on dry granules and tablets. Powder Technol..

[20] Mangal H., Kirsolak M., Kleinebudde P. (2016). Roll compaction/dry granulation: Suitability of different binders. Int. J. Pharm..

[21] NIPPON SODA General Information. NISSO HPC. https://www.nippon-soda.co.jp/hpc-e/care_stable.php.

[22] Europäische Arzneibuch-Kommission (2017). Europäisches Arzneibuch.

[23] Fell J.T., Newton J.M. (1970). Determination of tablet strength by the diametral-compression test. J. Pharm. Sci..

[24] Sun C.C., Hou H., Gao P., Ma C., Medina C., Alvarez F.J. (2009). Development of a high drug load tablet formulation based on assessment of powder manufacturability: Moving towards quality by design. J. Pharm. Sci..

[25] Li J., Tao L., Dali M., Buckley D., Gao J., Hubert M. (2011). The effect of the physical states of binders on high-shear wet granulation and granule properties: A mechanistic approach towards understanding high-shear wet granulation process. Part, I. PHYSICAL characterization of binders. J. Pharm. Sci..

[26] Dhenge R.M., Cartwright J.J., Doughty D.G., Hounslow M.J., Salman A.D. (2011). Twin screw wet granulation: Effect of powder feed rate. Adv. Powder Technol..

[27] Saleh M.F., Dhenge R.M., Cartwright J.J., Hounslow M., Salman A. (2015). Twin screw wet granulation: Binder delivery. Int. J. Pharm..

[28] Vanhoorne V., Bekaert B., Peeters E., De Beer T., Remon J.-P., Vervaet C. (2016). Improved tabletability after a polymorphic transition of delta-mannitol during twin screw granulation. Int. J. Pharm..

[29] Kumar A., Dhondt J., Vercruysse J., De Leersnyder F., Vanhoorne V., Vervaet C., Remon J.P., Gernaey K.V., De Beer T., Nopens I. (2016). Development of a process map: A step towards a regime map for steady-state high shear wet twin screw granulation. Powder Technol..

[30] Ai Q., Hounslow M.J., Salman A.D. (2018). Twin screw granulation: An evaluation of using micronized lactose as a solid binder. Chem. Eng. Res. Des..

[31] Portier C., De Vriendt C., Vigh T., Di Pretoro G., De Beer T., Vervaet C., Vanhoorne V. (2020). Continuous twin screw granulation: Robustness of lactose/MCC-based formulations. Int. J. Pharm..

[32] Ismail H.Y., Shirazian S., Singh M., Whitaker D., Albadarin A.B., Walker G.M. (2020). Compartmental approach for modelling twin-screw granulation using population balances. Int. J. Pharm..

[33] Seem T.C., Rowson N.A., Gabbott I., De Matas M., Reynolds G.K., Ingram A. (2016). Asymmetric distribution in twin screw granulation. Eur. J. Pharm. Biopharm..

[34] Dhenge R.M., Washino K., Cartwright J.J., Hounslow M.J., Salman A.D. (2013). Twin screw granulation using conveying screws: Effects of viscosity of granulation liquids and flow of powders. Powder Technol..

[35] Vercruysse J., Toiviainen M., Fonteyne M., Helkimo N., Ketolainen J., Juuti M., Delaet U., Van Assche I., Remon J.P., Vervaet C. (2014). Visualization and understanding of the granulation liquid mixing and distribution during continuous twin screw granulation using NIR chemical imaging. Eur. J. Pharm. Biopharm..

[36] Kumar A., Vercruysse J., Toiviainen M., Panouillot P.-E., Juuti M., Vanhoorne V., Vervaet C., Remon J.P., Gernaey K.V., De Beer T. (2014). Mixing and transport during pharmaceutical twin-screw wet granulation: Experimental analysis via chemical imaging. Eur. J. Pharm. Biopharm..

[37] Stoyanov E., Ehlig B., Tanev W. (2018). Eye on Excipients. Tablets and Capsules.

[38] Weatherley S., Thompson M., Sheskey P. (2012). A study of foam granulation and wet granulation in a twin screw extruder. Can. J. Chem. Eng..

[39] Thompson M., Mu B., Sheskey P. (2012). Aspects of foam stability influencing foam granulation in a twin screw extruder. Powder Technol..

[40] Takasaki H., Yonemochi E., Ito M., Wada K., Terada K. (2015). The importance of binder moisture content in Metformin HCL high-dose formulations prepared by moist aqueous granulation (MAG). Results Pharma Sci..

[41] Morkhade D.M. (2017). Comparative impact of different binder addition methods, binders and diluents on resulting granule and tablet attributes via high shear wet granulation. Powder Technol..

[42] Arndt O.-R., Baggio R., Adam A.K., Harting J., Franceschinis E., Kleinebudde P. (2018). Impact of different dry and wet granulation techniques on granule and tablet properties: A comparative study. J. Pharm. Sci..

